# *Lactobacillus reuteri* attenuated allergic inflammation induced by HDM in the mouse and modulated gut microbes

**DOI:** 10.1371/journal.pone.0231865

**Published:** 2020-04-21

**Authors:** Lingzhi Li, Zhifeng Fang, Xinyang Liu, Wenbin Hu, Wenwei Lu, Yuan-kun Lee, Jianxin Zhao, Hao Zhang, Wei Chen

**Affiliations:** 1 State Key Laboratory of Food Science and Technology, Jiangnan University, Wuxi, China; 2 School of Food Science and Technology, Jiangnan University, Wuxi, China; 3 National Engineering Research Center for Functional Food, Jiangnan University, Wuxi, China; 4 (Yangzhou) Institute of Food Biotechnology, Jiangnan University, Yangzhou, China; 5 Department of Microbiology & Immunology, Yong Loo Lin School of Medicine, National University of Singapore, Singapore, Singapore; 6 Wuxi Translational Medicine Research Center and Jiangsu Translational Medicine Research Institute Wuxi Branch, Wuxi, China; 7 Beijing Innovation Centre of Food Nutrition and Human Health, Beijing Technology and Business University (BTBU), Beijing, China; Telethon Institute for Child Health Research, AUSTRALIA

## Abstract

Gut microbiome plays an essential role in asthma development, and probiotic-based manipulation of the gut microbiome has been proposed to prevent asthma. Although the preventive effect of *Lactobacillus* supplementation against allergies has been reported, the precise *Lactobacillus* species beneficial for effective prevention of asthma remain unidentified and the underlying mechanisms remain unclear. Therefore, we aimed to investigate the efficacy of oral administration of six *Lactobacillus* species and the mechanism underlying asthma prevention via gut microbiome modulation. We investigated the effects of oral administration of *L*. *rhamnosus*, *L*. *fermentum*, *L*. *casei*, *L*. *gasseri*, *L*. *salivarius*, and *L*. *reuteri* (five strains of each species) on asthma and gut microbiome of house dust mite (HDM)-treated murine models of asthma. Of these, *L*. *reuteri* administration was the most effective: it alleviated airway inflammation, decreased total IgE and HDM-IgG1, and reduced Th2-associated pro-inflammatory cytokines. Moreover, modulation of specific microbial genera by *L*. *reuteri* was more effective in asthma prevention than the modulation of the overall microbiota composition. *Lactobacillus* and *Enterococcus* were enriched after *L*. *reuteri* supplementation and were closely associated with total IgE and IL-13 production. Furthermore, *L*. *reuteri* specifically altered the gut microbial function toward butyrate generation. Thus, *L*. *reuteri* may reduce the risk of asthma development by modulating specific gut microbiota to improve the lung immune environment. Our study suggests a novel option for gut microbiome manipulation via *L*. *reuteri* supplementation for suppression of asthma and other allergic diseases.

## Introduction

Allergies are immune-mediated disorders primarily caused by an IgE-dependent immunological reaction to an allergen (an innocuous environmental antigen). Depending on the allergen contact site, different clinical manifestations, characterized by the presence of IL-4, IL-5, IL-13, and IL-17A and increased levels of IgE and IgG, may occur in the gastrointestinal tract, skin, or airways [1–3]. Asthma is one such allergic disease, and it is defined as an allergen-mediated airway inflammatory disease [4], and among the most common affliction in the world [5]. The currently available treatment options alleviate the symptoms of asthma and other allergic diseases but cannot provide complete cure.

Intestinal microbiota is an important stimulatory factor for immune system development and function. The microbiota composition and metabolites in individuals with allergies have been reported to be different from those in healthy individuals [6–8]. Moreover, asthma involves gut microbiome perturbation and is associated with metabolic dysfunction. In mice, manipulation of the gut microbiome using oral probiotics or high-fiber dietary supplementation (which increases the synthesis of short-chain fatty acids (SCFA)) facilitates pro-resolving local and remote mucosal immunity [9,10]. Therefore, targeting the gut microbiota with probiotics, prebiotics, and dietary alteration would be a rational therapeutic approach to prevent asthma and other allergic diseases.

*Lactobacillus* is one of the most widely known probiotics. Low relative abundance of *Lactobacillus* was reported to be associated with asthma development early in life [11]. *Lactobacillus rhamnosus* GG was shown to be effective in the prevention of asthma in children at high risk [7], whereas *L*. *gasser*i was suggested to provide clinical benefits in school children with asthma [12]. However, *L*. *paracasei* supplementation did not ameliorate asthma in infants [13]. Thus, several studies have focused on the effectiveness of *Lactobacillus* in asthma, and *Lactobacillus* supplementation has been reported as an effective preventive strategy for allergy development in experimental and clinical studies. However, the precise *Lactobacillus* species that provide the essential beneficial effect and the underlying gut microbiome-dependent mechanisms remain unclear.

Therefore, in this study, we investigated the asthma-preventive effects of six *Lactobacillus* species, each constituting five strains. We aimed to assess the effectiveness of *Lactobacillus* against asthma and explore the mechanisms involved to better understand the immunomodulatory and preventive effects of probiotic in allergies.

## Materials and methods

### Bacterial strains

The study included 30 *Lactobacillus* strains belonging to six species: *L*. *rhamnosus*, *L*. *fermentum*, *L*. *casei*, *L*. *gasseri*, *L*. *salivarius*, and *L*. *reuteri*. The strains were isolated from food and fecal samples collected from healthy humans from several Chinese cities (Table 1). The stool samples were collected from the participants after obtaining a written informed consent from them or their legal guardians. All the strains were cultured in MRS medium at 37°C under static conditions. The bacterial cells were collected by centrifugation (6000 *g* for 15 min at 4°C), washed twice with sterile saline, and stored at −80°C until further use. Each candidate strain was adjusted to 10^9^ CFU. Five strains of the same species were mixed and administered orally to each mouse. The gradient dilution method was used to determine the number of bacterial cells.

**Table 1:** Strains used in animal experiments.

Group	Strain	Regional origin	Source
*L*. *rhamnosus*	JS-WX-24-1	Wuxi,Jiangsu Province,China	Infant feces
	JS-WX-3-L-2	Wuxi,Jiangsu Province,China	Infant feces
	TJ-DG-10-L10-6-1	Dagang,Tianjin Province,China	Infant feces
	H28L-1	Zhongxiang,Hubei Provice,China	Elder feces
	TJ-DG-9-L⑪10-5-1	Dagang,Tianjin Province,China	Infant feces
*L*. *fermentum*	HeNa-10-2-G-	Boai,Hennan Province,China	Elder feces
	TJ-DG-8-L⑨10-5-1b	Dagang,Tianjin Province,China	Infant feces
	13G-9	Rouergai,Sichuan Province,China	Human feces
	B76	Nantong,Jiangsu Province,China	Elder feces
	DL3-9	Meisan,Sichuan Province,China	Fermented milk
*L*. *casei*	M2-07-F01-L4-2-1	Rouergai,Sichuan Province,China	Human feces
	M2-06-F01-L4-2-3	Rouergai,Sichuan Province,China	cow dung
	CCFM1073 (JS-WX-3-L-3)	Wuxi,Jiangsu Province,China	Infant feces
	RS8-5	Meisan,Sichuan Province,China	Fermented milk
	FGDLZ41	Lianzhou,Guangdong Province,China	Infant feces
*L*.*gasseri*	lishouqian 3	Chengmai,Hainan Province,China	Elder feces
	C-1 A31	Boai,Hennan Province,China	Elder feces
	JS-WX-9-L-5	Wuxi,Jiangsu Province,China	Infant feces
	M2-C-F03-L-2	Rouergai,Xizang Province,China	Human feces
	AH-WH-7-4	Wuhu,Anhui Province,China	Elder feces
*L*. *salivarius*	GuXi-8-2-GMM	Bama,Guangxi Province,China	Elder feces
	GuXi-8-3-GMM	Bama,Guangxi Province,China	Elder feces
	GuXi-8-3-GMM	Bama,Guangxi Province,China	Elder feces
	GuXi-8-5-GMM	Bama,Guangxi Province,China	Elder feces
	4L-4	Enshi,Hubei Province,China	Elder feces
*L*. *reuteri*	CCFM1072 (FSDLZ13M6)	Laizhou,Shandong Province,China	Elder feces
	DYNDL2-16	Yunnan,Dali Province,China	Fermented milk
	CCFM1040 (YN-DL-1-3)	Yunnan,Dali Province,China	milk
	GDLZ10-5	Lianzhou,Guangdong Province,China	Child’s feces
	FZJTZ20M3	Taizhou,Zhejiang Province,China	Human feces

### Animal experiments

All animal experiments were performed according to the protocols approved by the Institutional Animal Ethics Committee of Jiangnan University (JN. No: 20170915b1920115 [11]) and were in compliance with the recommendations of national and international guidelines for the Care and Use of Laboratory Animals. All procedures were performed under anesthesia to minimize suffering.

**Fig 1:**
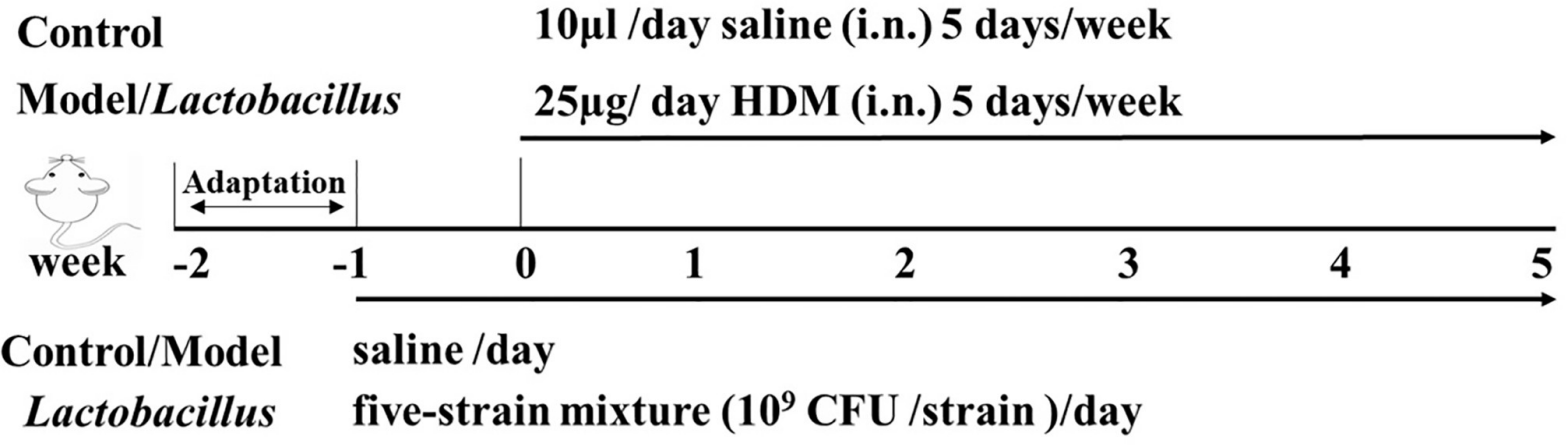
House dust mite (HDM) sensitization and exposure protocols. A timeline of HDM immunization and exposure and the administration of the six *Lactobacillus* species in the model has been provided.

Female BALB/c mice (aged 4–5 weeks) were obtained from Shanghai Experimental Animal Center (Shanghai, China) and housed in specific pathogen-free conditions. A standard diet and sterile filtered drinking water were provided ad libitum. The mouse model was developed as described previously [14]. Briefly, mice were intranasally exposed (i.n.) to purified 25 μg house dust mite (HDM) extract (Greer Laboratories Inc., Lenoir, NC, USA) dissolved in 10 μL saline for 5 days/week for 5 consecutive weeks. Control animals received 10 μL saline. After 1 week of acclimatization, the HDM-treated mice were randomly divided into seven groups (n = 6–7): the model group and six experimental groups. The control and model mice received the same volume of sterile saline every day. The six experimental group mice were orally administered the five-strain mixture of six different *Lactobacillus* species, starting 1 week before the first sensitization and continued till the end of the experiment. Allergic airway inflammation was analyzed on week 5 after the last challenge (Fig 1).

### Characterization of the allergic phenotype

To characterize the allergic phenotype, the following parameters were analyzed: (a) inflammation score in lung histology, (b) serum immunoglobulin, and (c) cytokines in BALF.

### Serum immunoglobulin analysis

Mouse serum was collected and frozen at −20°C. Serum immunoglobulins were measured using commercial ELISA kits: Mouse IgE ELISA kits (SenBeiJia Biotechnology Co., Ltd.) for total IgE; Mouse Serum Anti-HDM IgE and IgG1 Antibody Assay ELISA kits for HDM-specific IgE and IgG1 respectively (Chondrex, Inc., Washington USA); and Mouse HDM-IgG2a ELISA kit for HDM-specific IgG2a (Mlbio, Shanghai, China). Measurements were performed according to the manufacturers’ instructions.

### Lung cytokine analysis

Bronchoalveolar lavage was performed, and the bronchoalveolar lavage fluid (BALF) was collected and frozen at −20°C. Levels of IL-5, IL-13, and IL-17A in the BALF were measured using DuoSet ELISA kits (R&D Systems, Minneapolis, MN, USA) per the manufacturers’ recommendations.

### Lung histology

After sacrifice, left lobes of the mouse lungs were fixed in 4% paraformaldehyde and embedded in paraffin. Then, 5 μm-thick sections were obtained and stained with hematoxylin and eosin (H&E) and periodic acid–Schiff (PAS). Histological damage scores were assessed using a previously described system [15]. Four mice were randomly selected from each group to determine the degree of histological damage to the lung. The qualitative airway inflammation score of each mouse was on a 0–4 scale.

### Fecal sampling, DNA extraction, and amplicon sequencing

Feces from the colons of individual mice were collected in a sterile tube, snap frozen, and stored at –80°C until DNA extraction. DNA was extracted using a Fast DNA SPIN Kit for Feces (MP Biomedicals, Carlsbad, CA, USA). Then, 16S rDNA genes were PCR amplified using specific primers for the V3–V4 region of bacterial rDNA (341F and 806R) as described previously [16–18].

### Bioinformatics analyses

A data set consisting of 1,551,894 high-quality, classifiable read counts was generated via MiSeq sequencing analysis of 48 samples. All the sequences were clustered with representative sequences; 97% sequence identity cutoff was used. All data could undergo total sum scaling (TSS) normalization. Bacterial community diversity was analyzed using the R phyloseq [19] and vegan packages [20]. Alpha diversity (Shannon index)of the groups was compared using the Mann-Whitney/Kruskal-Wallis test (non-parametric); the data used for alpha diversity analysis were not rarefied. Hierarchical clustering was performed using the hclust function in the package stat of R. Linear discriminant analysis (LDA) effect size (LEfSe) was performed [21], and the LDA score was computed for taxa differentially abundant between two groups. |Log10 [LDA]| ≥ 2.0 and p < 0.05 (Kruskal–Wallis test) was considered to indicate statistical significance. Spearman’s rank test was performed for correlation analysis.

### SCFA analysis

SCFA levels in the cecal contents of mice were measured as described previously [22]. Briefly, cecal samples of approximately 50 mg were suspended with saturated NaCl and mixed thoroughly. SCFA were extracted using diethyl ether and quantified using a gas chromatograph equipped with a mass spectrometric detector (GCMS-QP2010 Ultra system, Shimadzu Corporation, Kyoto, Japan).

### Statistical analysis

Statistical analysis was performed using GraphPad Prism version 6 (La Jolla, CA, USA) and SPSS version 22.0 (SPSS Inc., Chicago, IL, USA). Results are presented as means with SEM. One-way ANOVA with Duncan’s new multiple range method was used to compare multiple groups. Differences between groups were considered significant at *p < 0.05.

## Results

### *L*. *reuteri* protects against airway inflammation in HDM-induced asthma model

**Fig 2:**
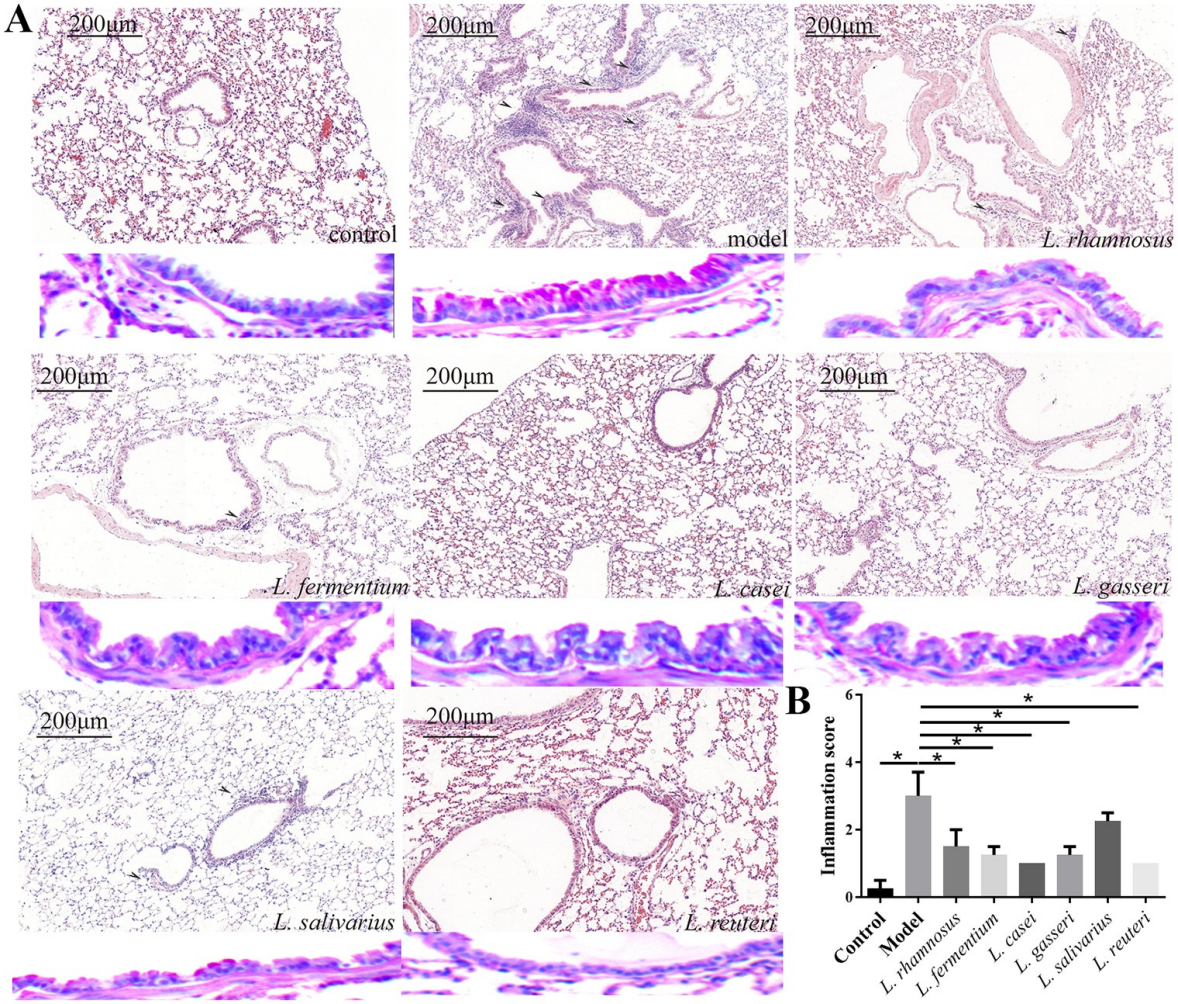
Ability of six *Lactobacillus* species to alleviate airway inflammation in house dust mite (HDM)-treated mice. (A) H&E-stained mouse lung sections showing airway inflammatory cell infiltrate; PAS-stained mouse lung sections showing goblet cell hyperplasia (red colour). (B) Inflammation scores for lung tissues (n = 4). *p<0.05.

In order to prove the impact of different *Lactobacillus* species on the development of airway inflammation, airway inflammatory cell infiltrate and goblet cell hyperplasia were assessed. For light microscopy and morphometry, mouse lung sections were stained with H&E and PAS to assess the inflammatory cell infiltrate and identify airway goblet cell hyperplasia, respectively. Histological analysis of lung sections revealed that *Lactobacillus* administration reduced airway inflammation in HDM-treated mice (Fig 2A). Moreover, the inflammation score (p < 0.05), a hallmark of airway inflammation, the higher the score, the more severe the inflammation, in all the *Lactobacillus* groups, except the *L*. *salivarius* group, was significantly lower than that of the model group. Compared to the control, the *L*. *fermentum*, *L*. *casei*, *L*. *gasseri*, and *L*. *reuteri* groups showed no significant difference in the inflammation score (Fig 2B).

### *L*. *reuteri* decreased Th2- and B cell-associated serum immunoglobulins

**Fig 3:**
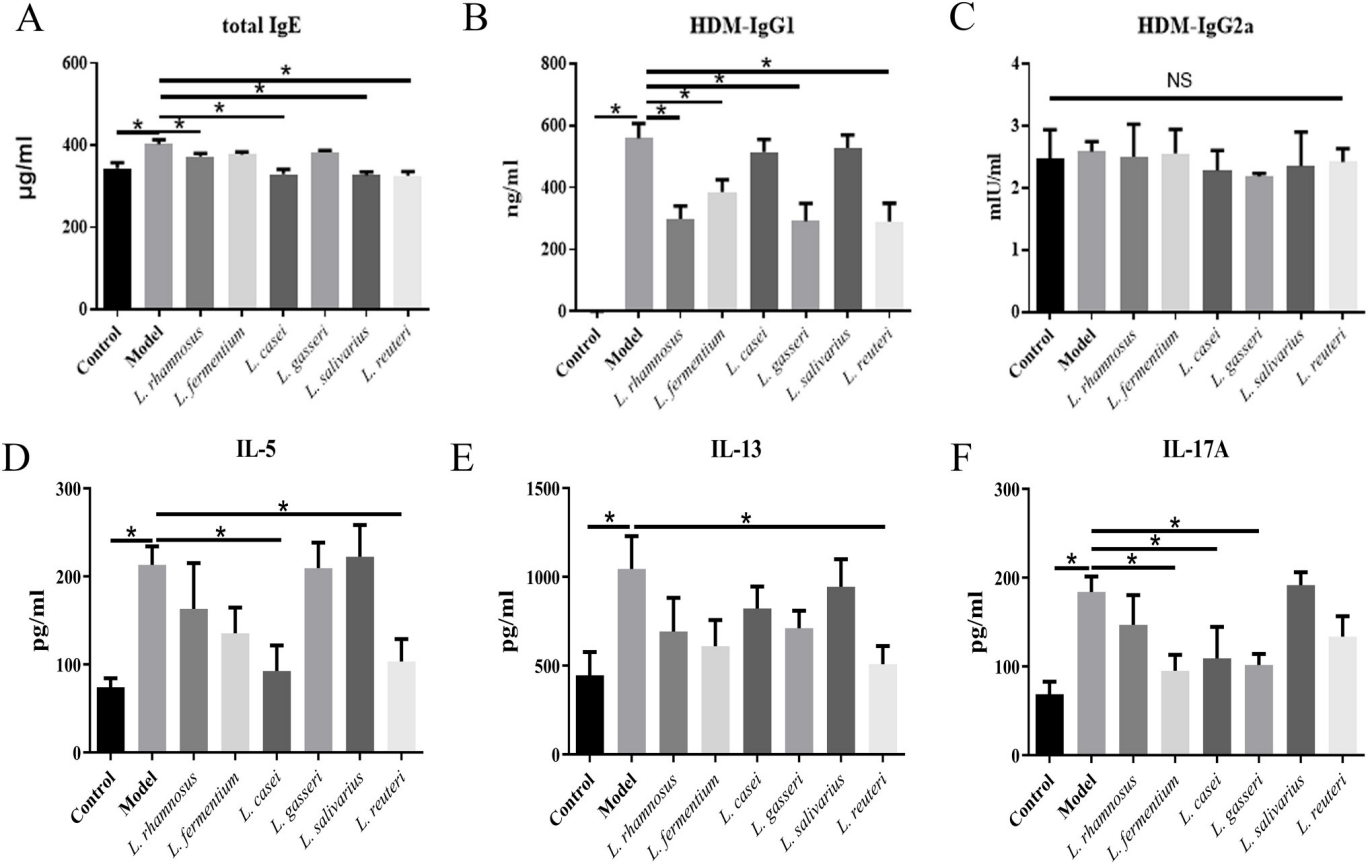
Ability of six *Lactobacillus* species to decrease asthma-associated phenotypes in house dust mite (HDM)-treated mice. (A–C) Serum levels of total IgE, HDM-specific IgG1, and HDM-specific IgG2a. (D–F) Cytokine production in the bronchoalveolar lavage fluid (BALF). *p<0.05.

The total IgE serum levels in the *L*. *rhamnosus*, *L*. *casei*, *L*. *salivarius*, and *L*. *reuteri* groups were significantly lower than those in the model group. Furthermore, compared to the control group, the *L*. *casei*, *L*. *salivarius*, and *L*. *reuteri* groups showed no significant difference in total IgE serum level. HDM-IgG1 levels in the *L*. *rhamnosus*, *L*. *fermentum*, *L*. *gasseri*, and *L*. *reuteri* groups were significantly lower (by 46.6%, 31.0%, 47.8%, and 47.9% respectively) than that in the model group. However, the HDM- IgG2a levels of the different groups were not significantly different (Fig 3A–3C).

### *L*. *reuteri* reduced Th2-based but not Th17-based inflammatory cytokine secretion in lungs

The secretory levels of IL-5, IL-13, and IL17A in the BALF from the HDM-treated model mice were significantly higher than those in the BALF from control mice. IL-5 levels were significantly reduced in the *L*. *casei* and *L*. *reuteri* groups, compared to the model group, but were not significantly different from those of the control group. The secretory levels of IL-13 were significantly decreased only in the *L*. *reuteri* group compared to the model group. Compared to the model group, the *L*. *fermentum*, *L*. *casei*, and *L*. *gasseri* groups showed reduced IL-17A levels (Fig 3D–3F).

Collectively, these results indicate that the different HDM-induced cytokine response in the lungs were significantly ameliorated in different *Lactobacillus* groups. *L*. *reuteri* administration ameliorated all the HDM-induced cytokine response in the lungs except IL-17A levels.

### *L*. *reuteri* altered the overall microbiota diversity in the colon of HDM-treated mice

**Fig 4:**
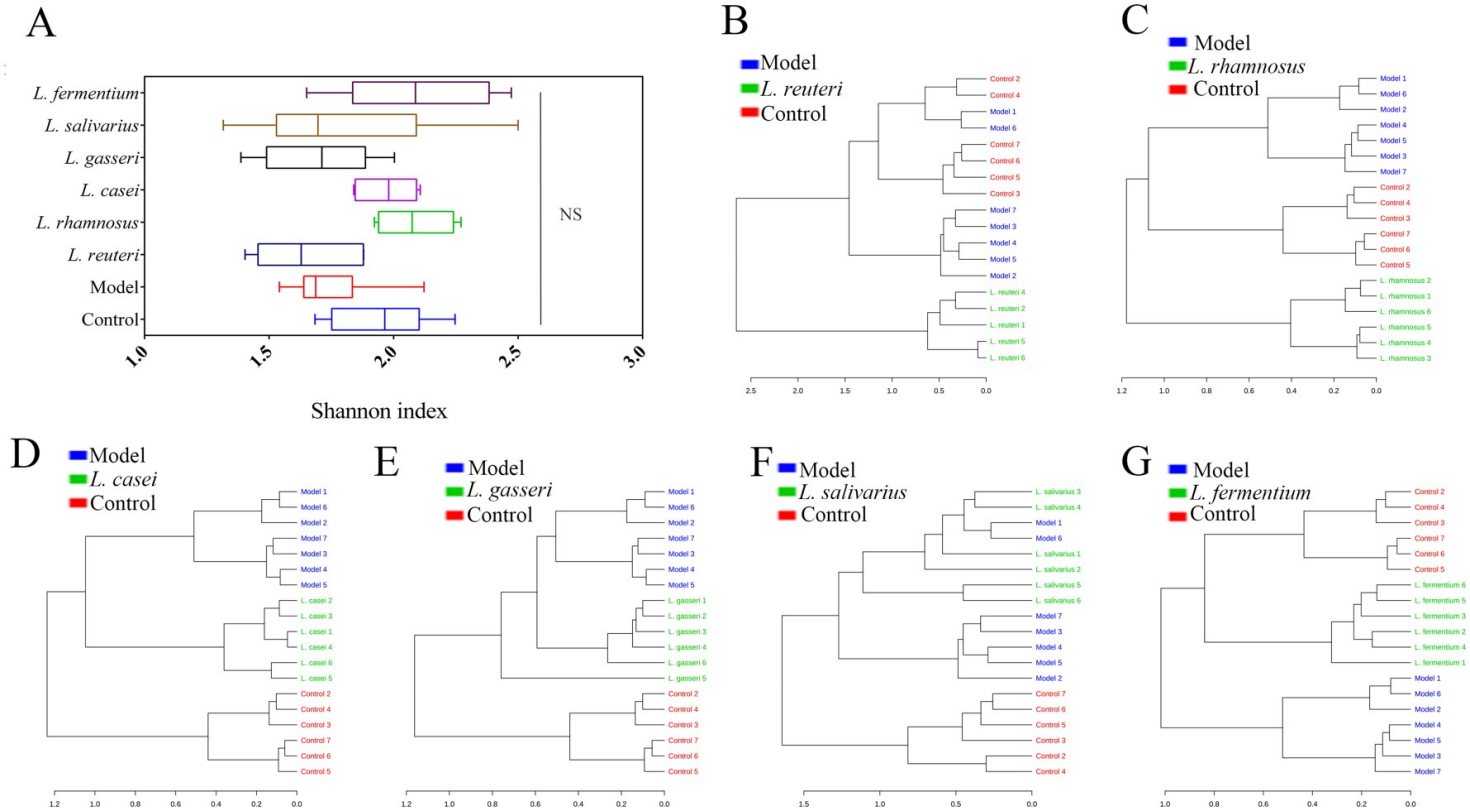
Taxonomic summary of gut microbiota in different mouse groups. (A) Boxplot for α-diversity measured using Shannon index at genus level. (B–G) Dendograms showing the results of cluster analysis. Distance was measured using Bray-Curtis distance and clustering algorithm using Ward’s method at the OTU level.

The overall microbiota diversity includes within-group and across-group microbial diversity. Within-group microbial diversity, Shannon index including the evenness and richness parameters, was not markedly different between the groups (Fig 4A). Furthermore, to assess across-group microbial diversity, hierarchical clustering was performed to obtain a dendrogram (Fig 4B–4G). Clustering analysis indicated that the bacterial profile in the model group was markedly different from that in groups subjected to *Lactobacillus* supplementation, except the *L*. *salivarius* group. The bacterial profiles in the *L*. *rhamnosus* and *L*. *reuteri* groups were considerably different from the microbiota profile of both control and model groups. Those in the *L*. *casei* and *L*. *gasseri* groups were different only from the model group. However, the bacterial profile in the *L*. *fermentum* group was considerably different from that in the model group and showed higher similarity with the bacterial profile of the control group.

### *L*. *reuteri*-treated increase in colonic *Lactobacillus* and *Enterococcus* was correlated with the improvement of the lung immune environment

**Fig 5:**
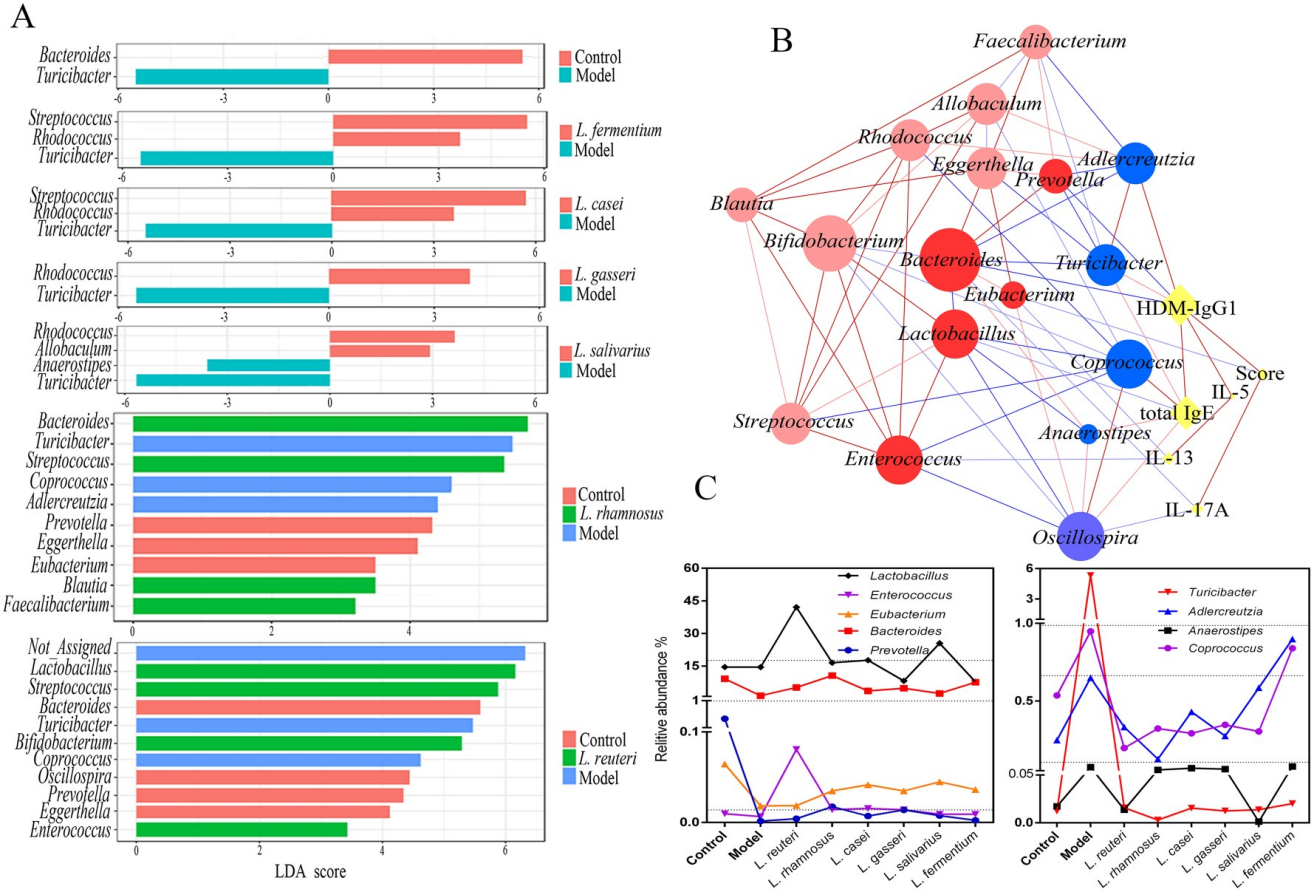
Gut microbiota analysis of house dust mite (HDM)-treated mice. (A) Linear discriminant analysis (LDA) score for gut microbiota from the control, model, *Lactobacillus reuteri*, *L*. *rhamnosus*, *L*. *casei*, *L*. *gasseri*, *L*. *salivarius*, and *L*. *fermentum* groups. (B) Network showing correlations between specific genera and asthma phenotype. Circle size is representative of the number of lines proportional to significant interactions. Red circles: ‘protective’ (dark color represents significant correlation with asthma phenotype); blue circles: ‘negative’; red lines: positive correlation; blue lines: negative correlation (light color represents significant correlation at the 0.05 level; dark color at the 0.01 level). (C) ‘protective’ and ‘negative’ relative abundance of altered bacterial genera in each *Lactobacillus* group.

At the genus level, the control and model groups showed a marked difference in the abundance of two bacterial taxa. Compared to the controls, the models showed significantly reduced *Bacteroides* but considerably increased *Turicibacter* (Fig 5A).

Using the results from hierarchical clustering, we categorized the *Lactobacillus* groups into three classes for further LEfSe analysis. We found that 17 taxa sequences were significantly altered in the *Lactobacillus* groups. *Rhodococcus*, *Streptococcus*, *Allobaculum*, *Blautia*, *Faecalibacterium*, *Lactobacillus*, *Bifidobacterium*, and *Enterococcus* increased whereas *Turicibacter*, *Anaerostipes*, *Coprococcus*, and *Adlercreutzia* decreased in the *Lactobacillus* groups, compared with the model group. The abundance of *Bacteroides*, which decreased in the model group, increased only after *L*. *rhamnosus* administration. *Prevotella*, *Eggerthella*, *Eubacterium*, and *Oscillospira* were enriched in control (Fig 5A).

Global integration using Spearman correlation for all asthma phenotypes with the 17 microbial genera revealed that ten microbial genera were associated with the asthma phenotypes, except IL-5 levels (Fig 5B). The bacteria that were enriched in the model group and showed the highest value for positive correlation with asthma phenotypes included *Turicibacter* and total IgE (ρ = 0.429, P = 0.010) and HDM-IgG1 (ρ = 0.342, P = 0.039); *Anaerostipes* and total IgE (ρ = 0.328, P = 0.047); *Coprococcus* and total IgE (ρ = 0.485, P = 0.002); and *Adlercreutzia* and HDM-IgG1 (ρ = 0.517, P = 0.001). Significant negative correlations observed in the *Lactobacillus* groups included *Lactobacillus* and total IgE (ρ = -0.414, P = 0.011) and *Enterococcus* and IL-13 (ρ = -0.365, P = 0.012), and those in the controls included *Bacteroides* and IL-17A (ρ = -0.362, P = 0.012); HDM-IgG1 (ρ = -0.553, P = 0.001); *Prevotella* and HDM-IgG1 (ρ = -0.470, P = 0.004); *Eubacterium* and IL-13 (ρ = -0.349, P = 0.016); and *Eubacterium* and score (ρ = -0.391, P = 0.033). Interestingly, *Oscillospira* was positively correlated with total IgE (ρ = 0.385, P = 0.019) and negatively correlated with IL-17A (ρ = -0.362, P = 0.012).

Except *Lactobacillus* and *Enterococcus*, eight other microbial genera differed between the control and model groups. The abundance of *Lactobacillus* and *Enterococcus* increased simultaneously only after *L*. *reuteri* administration (Fig 5C).

### *L*. *reuteri* altered metabolic function and increases butyrate in mouse cecum

**Fig 6:**
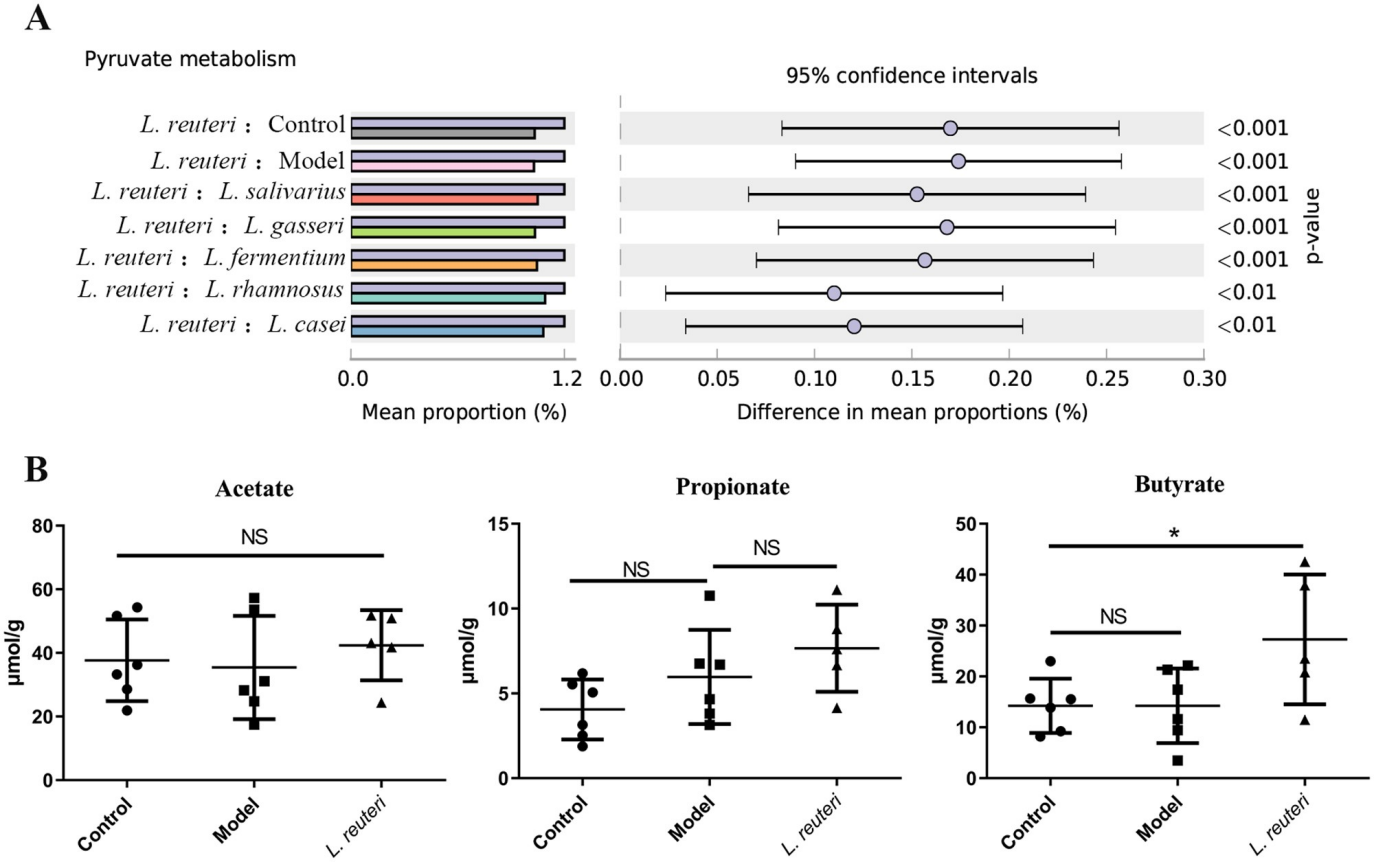
Effect of *Lactobacillus reuteri* supplementation on short-chain fatty acid (SCFA) production. (A) A significant occurrence of difference pyruvate metabolism between *L*. *reuteri* and other groups. (B) Acetate, propionate, and butyrate levels in cecum contents of mice.

Microbial functions were predicted using PICRUSt. Compared with the model and control groups, the *L*. *reuteri* group showed significant changes in the levels of 153 metabolites. Pyruvate metabolism, the classic pathway for the SCFA formation, was the most significantly altered pathway in the *L*. *reuteri* group, compared to the other groups (Fig 6A). Cecal contents were analyzed to confirm whether *L*. *reuteri* regulated gut microbiota to increase SCFA production. Compared to the model group, the *L*. *reuteri* group showed significant increase in cecal butyrate levels but not in cecal acetate and propionate levels (Fig 6B).

## Discussion

The aim of this study was to investigate the preventive effect of different *Lactobacillus* species on modulation of gut microbiota for asthma. Using an HDM-treated murine model for asthma, we demonstrate the preventive effects of edible *Lactobacillus* strains against the development of Th2 and Th17-driven allergic inflammation. Although the different *Lactobacillus* species showed varying degrees of attenuation of the HDM-induced allergic inflammation, our results reveal that continuous *Lactobacillus* supplementation could regulate immune function in mice and impart an anti-inflammatory effect in the lung. Of the six *Lactobacillus* species tested, *L*. *reuteri* showed better efficacy in ameliorating asthma phenotypes.

All *Lactobacillus* species investigated in this study could modulate the overall gut microbiota. *L*. *fermentum* supplementation showed recovery of the gut microbiota from that observed in the models to that observed in the controls; however, it did not show the best preventive effect against asthma. It has been speculated that specific microbes might be more important than overall microbial diversity in association with allergic diseases [8].

In this study, specific microbes modulated by the different *Lactobacillus* species were similar. This showed similar patterns of alteration in the microbiota composition, suggesting the same role of these *Lactobacillus* species in asthma pathogenesis. Therefore, we further speculate that these *Lactobacillus* species with different ability may occupy the same ecological niche in the gut. However, few studies have focused on the functional properties of specific microbial genera in allergic diseases, including asthma.

In this study, we performed Spearman correlation test to evaluate the relations among specific genera and asthma phenotypes. The specific genera enriched in the *Lactobacillus* groups showed positive correlation with the specific genera enriched in the control, and negative correlation in the model and with asthma phenotypes. They could be divided into ‘protective’ (more abundant in *Lactobacillus* groups and control group) and ‘negative’ (more abundant in the model group) genera.

*Lactobacillus*, *Bifidobacterium*, *Enterococcus*, and *Streptococcus* are widely considered as probiotics [23]. *Lactobacillus* and *Bifidobacterium* are beneficial in reducing allergic diseases [24,25]. *Rhodococcus* and *Enterococcus* exhibit anti-inflammatory effects in sensitized animal models [26,27]. Furthermore, *Bacteroides* were found to be more abundant in healthy adults than in asthmatic children [1,28], and *Streptococcus* abundance decreased in children with food sensitization in early life [29]. Moreover, inflammation has been suggested to provide a favorable environment for *Turicibacter* [30,31], thereby implicating the role of *Turicibacter* in disrupting the gut microbiota and influencing asthma phenotypes.

These results support the surmise that specific genera contribute to allergic sensitization and are ‘protective’ or ‘negative’ with respect to asthma initiation. This may be one reason underlying the preventive effect of *Lactobacillus* against asthma. The ‘protective’ bacteria can act against the ‘negative’ bacteria via microbial exclusion from the mucosa (an ecological effect) [32]. However, further studies are warranted to confirm these possible causal relations.

Of all the *Lactobacillus* species investigated in this study, *L*. *reuteri* showed the strongest effect against asthma. Increase in the abundance of *Lactobacillus* and *Enterococcus* by *L*. *reuteri* administration may reduce asthma risk. Peculiarly, the abundance of these two bacteria was not markedly different between other *Lactobacillus* groups and model mice. *Lactobacillus* was the only specific genera that was strongly negatively associated with the level of total IgE, the biomarker for allergy [33]. Moreover, it was the most abundant among the ‘protective’ bacteria and was significantly increased by three folds after *L*. *reuteri* supplementation. Because *Lactobacillus* colonizes the intestinal tract, it may be considered an effective probiotic with properties not restricted to the gut. Pathway analysis indicated that *L*. *reuteri* specifically treated a functional shift towards SCFA production in the gut. We observed an increase in butyrate content in the gut only after *L*. *reuteri* supplementation. The anti-inflammatory role of butyrate has been reported previously [34]. In fact, *Enterococcus* and *Streptococcus* are butyrate-producing bacteria. In addition, *Lactobacillus* and *Bifidobacterium* also play an important role in the increase of butyrate production: they are able to produce the acetate and lactate, which are used to synthesize butyrate. Therefore, the upregulation of butyrate in *L*. *reuteri* group observed in our study is important for the preventive effect against asthma and may be associated with the increase in *Lactobacillus*, *Bifidobacterium*, *Enterococcus* and *Streptococcus* abundance.

In this study, we demonstrated the effectiveness of *Lactobacillus* against asthma. In addition to modulating the common microbiome of *Lactobacillus*, *L*. *reuteri* also modulated specific gut microbial genera, thereby providing a better protective effect against asthma. *L*. *reuteri* may be considered as a potential candidate when screening for Lactobacilli with preventive effect against asthma. Because the reciprocal interaction of *Lactobacillus* supplementation with the gut microbiota is complex, further research involving different *L*. *reuteri* strains is essential to elucidate the mechanisms underlying the preventive role of *Lactobacillus*.

## Conclusions

To our knowledge, this is the most large-scale report in which the preventive effects of different *Lactobacillus* species on allergies are investigated in a murine HDM-induced asthma. Our study showed that *L*. *reuteri* supplementation was more efficient than the five other species investigated in this study for asthma prevention. The modulation of specific gut microbes by *L*. *reuteri* altered the gut microbial function toward increased butyrate production, which alleviated airway inflammation and the Th2 response in lung tissues. Therefore, such modulation is more effective for asthma prevention than the modulation of overall gut microbiota composition. Thus, targeting the gut microbiota via *L*. *reuteri* supplementation may be considered as an ideal strategy for the prevention of asthma and other allergic diseases.
